# Phenotypic and Epigenetic Adaptations of Cord Blood CD4+ T Cells to Maternal Obesity

**DOI:** 10.3389/fimmu.2021.617592

**Published:** 2021-04-12

**Authors:** Suhas Sureshchandra, Norma Mendoza, Allen Jankeel, Randall M. Wilson, Nicole E. Marshall, Ilhem Messaoudi

**Affiliations:** ^1^ Department of Molecular Biology and Biochemistry, School of Biological Sciences, University of California Irvine, Irvine, CA, United States; ^2^ Institute for Immunology, University of California Irvine, Irvine, CA, United States; ^3^ Division of Biomedical Sciences, University of California Riverside, Riverside, CA, United States; ^4^ Maternal-Fetal Medicine, Oregon Health & Science University, Portland, OR, United States; ^5^ Center for Virus Research, University of California Irvine, Irvine, CA, United States

**Keywords:** pregravid obesity, neonates, umbilical cord blood CD4+ T cells, DNA methylation, chromatin accessibility, transcription

## Abstract

Pregravid obesity has been shown to disrupt the development of the offspring’s immune system and increase susceptibility to infection. While the mechanisms underlying the impact of maternal obesity on fetal myeloid cells are emerging, the consequences for T cells remain poorly defined. In this study, we collected umbilical cord blood samples from infants born to lean mothers and mothers with obesity and profiled CD4 T cells using flow cytometry and single cell RNA sequencing at resting and following *ex vivo* polyclonal stimulation. We report that maternal obesity is associated with higher frequencies of memory CD4 T cells suggestive of *in vivo* activation. Moreover, single cell RNA sequencing revealed expansion of an activated subset of memory T cells with maternal obesity. However, *ex vivo* stimulation of purified CD4 T cells resulted in poor cytokine responses, suggesting functional defects. These phenotypic and functional aberrations correlated with methylation and chromatin accessibility changes in loci associated with lymphocyte activation and T cell receptor signaling, suggesting a possible link between maternal obesogenic environment and fetal immune reprogramming. These observations offer a potential explanation for the increased susceptibility to microbial infection in babies born to mothers with obesity.

## Introduction

High pre-pregnancy (pregravid) body mass index (BMI) is associated with poor health outcomes for both the mother and the offspring. In particular, babies born to mothers with obesity are more susceptible to infectious diseases (1–3), with increased risks for bacterial sepsis and necrotizing enterocolitis that require admission to the neonatal intensive care unit (4, 5). Additionally, offspring of mothers with obesity exhibit increased susceptibility to respiratory diseases (wheezing and childhood asthma (6–8)), cancer (9), and metabolic diseases (type-2 diabetes and cardiovascular diseases) (3).

Similarly, experimental animal model studies where maternal obesity was induced using high fat diet (HFD) exposure during gestation showed poor disease outcomes in the offspring following exposure to respiratory syncytial virus (RSV) (10) and bacterial infections (2). Furthermore, pups born to obese dams exhibit greater susceptibility to autoimmune and inflammatory diseases, and a shift from IgG to IgE antibody responses following vaccination with ovalbumin (1, 2). Additionally, lamina propia lymphocytes isolated from pups born to dams fed a western style diet (WSD) during gestation produced a larger IL1β and IL-17 response (2). This heightened inflammatory response is in line with enhanced offspring susceptibility to DSS-induced colitis (11). In contrast, splenocytes from pups born to obese dams generated a dampened response following lipopolysaccharide (LPS) stimulation in comparison to cells from pups born to control-diet fed dams (2). Finally, fewer regulatory T cells (Tregs) were reported in both the spleen and the gut (2). Collectively, these clinical and experimental observations suggest that pregravid obesity disrupts development and maturation the offspring immune system in a tissue and cell specific.

Mechanisms underlying dysregulated immunity in the offspring are beginning to emerge (12). Recent studies from our laboratory demonstrated a dampened response of human umbilical cord blood (UCB) monocytes obtained from babies born to mothers with a pregravid BMI>30 following LPS stimulation (13). This dysregulation was cell intrinsic and partially explained by epigenetic reprogramming (14). We also reported a reduced frequency of UCB CD4 T cells as well as IL-4-secreting UCB CD4 T cells but no differences in CD8 T cell responses in samples obtained from babies born to mothers with obesity (13). However, these studies were performed using mixed populations of umbilical cord blood mononuclear cells, hence it is still unclear if defects in CD4 T cell responses are intrinsic.

Therefore, in this study, we sought to investigate the impact of maternal pregravid BMI on UCB CD4 T cell responses to *ex vivo* stimulation and the underlying epigenetic changes. Our analysis shows that maternal obesity leads to increased accumulation of effect or memory UCB CD4 T cells that was associated with increased methylation of loci associated with “naïve” T cell identity in samples from babies born to mothers with obesity. Despite the higher frequency of effector memory cell, UCB CD4 T cells from the obese group responded poorly to both CD3/CD28 and PMA/Ionomycin stimulation in terms of cytokine production and transcriptional changes. This dampened response was in line with a reduction in chromatin accessibility at loci associated with TCR and insulin signaling. Taken together, these results support the hypothesis that maternal pregravid obesity alters the development and epigenome of CD4 T cells in the offspring, providing a potential link to increased susceptibility to chronic inflammatory diseases and infection.

## Materials and Methods

### Human Subjects – Cohort Descriptions and Ethics Approval

This research project was approved by the Institutional Ethics Review Boards (IRB) of Oregon Health and Science University, University of California, Riverside and University of California Irvine. All subjects provided signed consent before enrolling in the study. Phenotyping and intracellular responses of cord T cells were confirmed in two independent cohorts. Cohort I (recruited at OHSU) consisted of 34 lean mothers mean age of 31.25 ± 4.9 years and a pre-pregnancy BMI of 21.8 ± 1.9 kg/m^2^ (lean) and 16 mothers with obesity with a mean age of 30.5 ± 5.6 and a pre-pregnancy BMI of 36.6 ± 4.5 kg/m^2^. Cohort II (recruited at UCR) consisted of 17 lean mothers mean age of 30.70 ± 4.2 years and a pre-pregnancy BMI of 22.6 ± 2.2 kg/m^2^ (lean) and 11 mothers with obesity with a mean age of 35.5 ± 5.3 and a pre-pregnancy BMI of 38.1 ± 6.7 kg/m^2^. Cord blood samples from term babies (both vaginal and C-section) delivered to 15 lean and 11 obese subjects were used for genomic experiments described in this study. Only non-smoking women without gestational diabetes or gestational hypertension who had an uncomplicated singleton gestation were included in this study.

### Sample Collection and Processing

Complete blood counts were obtained by Beckman Coulter Hematology analyzer (Brea, CA). Umbilical cord blood mononuclear cells (UCBMC) and plasma were obtained by standard density gradient centrifugation over Ficoll (GE Healthcare). UCBMC were frozen in 10% DMSO/FBS using Mr. Frosty Freezing Containers (Thermo Fisher, Waltham MA), and stored in liquid nitrogen until analysis. Plasma was stored at -80C until analysis.

### UCBMC Phenotyping

10^6^ UCBMC (n= 51 lean, 27 obese) were washed with PBS and stained using the following cocktail of antibodies: PB-CD4, ECD-CD8b, PE-CD19, PCP-CY5.5-CD45RA, APC-CCR7 to delineate naïve and memory T cell populations. All samples were acquired with the Attune NxT Flow Cytometer (ThermoFisher Scientific, Waltham MA) and analyzed using FlowJo 10.5.0 (Ashland OR).

### Intracellular Cytokine Staining

For T cell responses, 10^6^ UCBMC (n=39 lean, 17 obese) were stimulated for 16h at 37°C in RPMI supplemented with 10% FBS in the presence or absence of anti-CD3/CD28 dynabeads per manufacturer’s instructions (ThermoFisher Scientific); Brefeldin A (Sigma, St. Louis MO) was added after 1-hour incubation. Cells were stained for surface markers PB-CD4, ECD-CD8, fixed, permeabilized, and then stained intracellularly for APC-TNFα, PE-Cy7-IFNγ, FITC-IL-4, and AF700-IL-2. All samples were acquired with the Attune NxT Flow Cytometer (ThermoFisher Scientific, Waltham MA) and analyzed using FlowJo 10.5.0 (Ashland OR). Group differences were tested using unpaired t-test followed by welch’s correction on Prism 8 (GraphPad, San Diego CA).

### Cell Sorting and Single Cell RNA-Seq Library Generation

Frozen UCBMC (n=2/group) from a fourth independent cohort were thawed and enriched for live cells using SYTOX Blue (1:1000, ThermoFisher) on the BD FACS Aria Fusion into RPMI (supplemented with 30% FBS). Sorted cells were counted in triplicates and resuspended in PBS with 0.04% BSA in a final concentration of 1200 cells/uL. Cells were immediately loaded in the 10X Genomics Chromium Controller with a loading target of 17,600 cells. Libraries were generated using the V3 chemistry per manufacturer’s instructions (10X Genomics, Pleasanton CA). Libraries were sequenced on Illumina NovaSeq with a sequencing target of 50,000 reads per cell.

### Single Cell RNA-Seq Data Analysis

Raw reads were aligned and quantified using the Cell Ranger Single-Cell Software Suite (version 3.0.1, 10X Genomics) against the GRCh38 human reference genome using the STAR aligner. Downstream processing of aligned reads was performed using Seurat (version 3.1.1). Droplets with ambient RNA (cells fewer than 400 detected genes), potential doublets (cells with more than 4000 detected genes), dying cells (cells with more than 20% total mitochondrial gene expression) were excluded during initial QC. Data objects from lean and obese group were integrated using Seurat. Data normalization and variance stabilization were performed using *SCTransform* function using a regularized negative binomial regression, correcting for differences in mitochondrial and ribosomal gene expression levels and stage of cell cycle. Dimension reduction was performed using *RunPCA* function to obtain the first 30 principal components followed by clustering using the *FindClusters* function in Seurat. Visualization of clusters was performed using Seurat’s UMAP implementation in *runUMAP* function. Cell types were assigned to individual clusters using *FindMarkers* function with a log fold change cutoff of at least 0.5 (≥0.5 or ≤-0.5) and using published markers for human PBMC.

UMAP clusters of cells with high *IL7R* and *CD3* (*CD3D*, *CD3E*) expression but low *CD8A* and *CD8B* expression were extracted from Seurat object using the *subset* function. Cells were re-clustered and visualized until all clusters of doublets, dead cells and CD8 T cells were removed. Level of *CCR7* expression was used to classify naïve/central memory from effector memory T cells while high *FOXP3* and *CTLA4* classified cells as Tregs. Differential gene expression within subsets of T cells between lean and obese group was tested using Wilcoxon rank sum test followed by bonferroni correction. Two-way functional enrichment of differential signatures was performed on Metascape. Differential hierarchies within the T cell compartment were reconstructed using Monocle (version 2.8.0). Briefly, clustering was performed using t-SNE and differential genes identified using Monocle’s *differentialGeneTest*. These genes were used for ordering cells on a pseudotime. Violin plots and bubble plots were generated using ggplot2 in R.

### Stimulation of Purified CD4 T Cells

Frozen UCBMCs were thawed (n=3 lean, 3 obese) and CD4 T cells were purified using antibodies conjugated to magnetic microbeads per manufacturer’s recommendations (Miltenyi Biotech, San Diego CA). Purity was assessed using flow cytometry and was ≥90% for all samples. Purified UCB CD4 T cells were plated at 1x10^5^/well and stimulated with 1 ug/mL PMA and 10 ug/mL Ionomycin for 16 hours at 37C and 5% CO_2_. Following the 16hr incubation, cells were spun down and pellets were resuspended in Trizol (Qiagen) for RNA extraction.

For stimulation of naïve CD4 T cells, frozen UCBMCs were thawed (n=6/group), stained with PerCP-Cy5.5-CD4, PB-CD95, and FITC-CD28, and live naïve (CD4+CD28+CD95-) cells were purified using the BD FACS Aria Fusion into RPMI supplemented with 30% FBS. Cells were washed, counted, plated at a density of 60,000 cells/well and then stimulated with 1 ug/mL PMA and 10 ug/mL Ionomycin for 16 hours. Cells were spun down and supernatants stored at -80C for future analyses. Cell pellets were stained with APC-Cy7-CD25, BV605-CD3, and PE-CD69 for 30 minutes, washed twice, acquired with the Attune NxT Flow Cytometer (ThermoFisher Scientific, Waltham MA). Samples were analyzed using FlowJo 10.5.0 (Ashland OR). Differences in median fluorescence intensity in response to stimulation and between groups was tested using ordinary one-way ANOVA followed by Holm-Sidak’s multiple comparison test on Prism 8 (GraphPad, San Diego CA).

### Luminex Assays

Cytokines, chemokines, and growth factors in undiluted cell culture supernatants and diluted UCB plasma (1:1) were measured using a human 37-plex luminex panel R & D Systems, Minneapolis MN). Metabolic hormones were measured using a 3-plex kit measuring insulin, leptin, and PYY (Millipore, Burlington MA). Adipokines were assayed using a 5-plex kit measuring adiponectin, adipsin, lipocalin-2, total PAI-1, and resistin (Millipore, Burlington MA). Samples were run in duplicates on the Magpix instrument (Luminex, Austin TX). Data were fit using a 5P-logistic regression on the xPONENT software. Values below the limit of detection were designated as half of the lowest limit. Data in pg/mL were tested for normality using Shapiro-Wilk test. Statistical differences in plasma proteins were tested using unpaired t-test with welch’s correction. Differences in protein in supernatants were tested using ordinary one-way ANOVA followed by Holm-Sidak’s multiple comparison tests. All statistical tests were performed on Prism 8 (GraphPad, San Diego, CA).

### RNA Extraction and Library Preparation

RNA from CD4 T cell pellets (n=3 lean, 3 obese) following PMA/ionomycin stimulation was extracted using mRNeasy kit (Qiagen, Valencia CA). RNA concentration and integrity were determined using Agilent 2100 Bioanalyzer. Libraries were generated using TruSeq Stranded Total RNA-Seq kit (Illumina, San Diego CA). Following rRNA depletion, mRNA was fragmented, converted to double stranded cDNA and adapter ligated. Fragments were enriched by PCR amplification and purified. Library concentrations were measured using Qubit; library quality and peak sizes were assessed on the Bioanalyzer. Libraries were multiplexed and sequenced on the HiSeq4000 platform to yield an average 20 million 100 bp single end reads.

### RNA-Seq Analysis

Data QC was performed retaining bases with quality scores ≥20 and reads ≥35 base pair long. Reads were aligned to the human genome (hg19) using splice aware aligner TopHat using annotations available from ENSEMBL repository. Read quantification was performed using GenomicRanges package in R and normalized using RPKM method. Responses to PMA/Ionomycin were modeled pair-wise for every sample using negative binomial GLMs following low read count filtering (median RPKM ≤5) using edgeR. Genes with expression difference (FDR < 0.05) and log_2_ fold change (≥ 1.5 or ≤ -1.5) were considered as differentially expressed genes (DEG). Gene expression data have been deposited in NCBI’s Sequence Read Archive.

### Methyl-Seq Library Generation

200 ng genomic DNA was isolated from resting 2-5x10^5^ CD4+ T cells (n= 6 lean, 4 obese) using *Quick-gDNA* MiniPrep (Zymo Research, Irvine CA), sheared to 100-200 bp using Covaris Ultrasonicator (Covaris, Woburn MA) and verified using Agilent 2100 Bioanalyzer. DNA cytosine methylation levels in purified resting UCB CD4 T cells were measured at single base resolution using a targeted bisulfite sequencing approach (SureSelectXT Human Methyl-Seq enrichment system, Agilent, Santa Clara CA), focusing on regions where methylation is known to impact gene regulation (cancer tissue-specific differentially methylated regions or DMRs, GENCODE promoters, CpG islands, shores and shelves ±4kb, DNase I hypersensitive sites and RefGenes). The ends of sheared DNA were repaired, 3’ adenylated, and ligated with methylated adapters. These DNA fragments were then hybridized with 120 nt biotinylated RNA library fragments that recognize methylated DNA regions and isolated using streptavidin beads followed by bisulfite conversion using Zymo EZ DNA Methylation-Gold Kit (Zymo, Irvine CA), which converts unmethylated cytosines to uracils. Libraries were then PCR amplified, ligated with unique indices, and sequenced on Illumina NextSeq500 platform to generate 60 million 50 bp paired-end reads per sample. Efficiency of bisulfite conversion was measured using 20 pg of unmethylated phage lambda DNA spiked in with each sample before DNA fragmentation. The bisulfite non-conversion rate was calculated as the percentage of cytosines sequenced at cytosine reference positions in lambda genome.

### Methyl-Seq Analysis

Raw reads were assessed for quality and trimmed to ensure bases with quality scores less than 30 and reads shorter than 50 bases were eliminated. QC passed reads were aligned to the human genome hg19 using Bismark. To measure bisulfite conversion rates, reads were mapped to phage lambda genome that was spiked into each library. PCR duplicates in the alignment files were filtered using picard tools and file conversions were performed using samtools. Single base resolution methylation calls were made using the R package methylKit. Differences in methylation between lean and obese groups was measured using a logistic regression model built in methylKit, allowing us to identify differentially methylated cytosines (DMCs) with at least 25% difference in methylation levels and an FDR corrected p-value of at least 0.05. DMCs overlapping chromosomes X, Y and mitochondrial genome were eliminated from subsequent analyses. We performed optimized region analysis of methylation using eDMR (https://github.com/ShengLi/edmr), which uses a bimodal distribution to identify accurate boundaries of regions/loci harboring significant epigenetic changes without *a priori* assignment of DMR length. DMRs were annotated using ChIP-Seeker using annotations available for hg19 on UCSC database.

### ATAC-Seq Library Generation

Chromatin accessibility was profiled using bulk ATAC-Seq as originally described (15). Briefly, 1x10^5^ purified CD4+ T cells (n=2/group) were lysed in lysis buffer containing 10 mM Tris-HCl (pH 7.4), 10 mM NaCl, 3 mM MgCl2, and NP-40 for 10 min on ice to prepare the nuclei. Immediately after lysis, nuclei were spun at 500g for 5 min to remove the supernatant. Nuclei were then incubated with transposition mixture (100 nM Tn5 transposase, 0.1% Tween-20, 0.01% Digitonin and TD buffer) at 37C for 30 min. PCR was performed to amplify the library for 6-10 cycles using the following PCR conditions: -72C for 3 min; 98C for 30s and thermocycling at 98C for 10s, 63C for 30s and 72C for 1 min. After PCR reaction, libraries were purified with the 1.2X AMPure beads. Libraries were quality checked on the Bioanalyzer, multiplexed and sequenced on 75 bp paired ended on NextSeq500 (Illumina San Diego CA).

### ATAC-Seq Analysis

Paired reads from sequencing were quality checked using FASTQC and trimmed to retain reads with quality scores of ≥20 and minimum read lengths of 50 bp. Trimmed paired reads were aligned to the human genome (hg38) using Bowtie2 (–X 2000 –k 1 –very-sensitive –no-discordant –no-mixed). Reads aligning to mitochondrial genome and allosomes were removed using samtools. PCR duplicate artifacts were then removed using Picard. Finally, poor quality alignments and improperly mapped reads were filtered using samtools (samtools view –q 10 –F 3844). To reflect the accurate read start site due to Tn5 insertion, BAM files were repositioned using ATACseqQC package in R. Positive and negative strands were offset by +4bp and -5bp respectively. Samples within a group were merged and sorted using samtools.

Sample QC and statistics for merged BAM files were generated using HOMER makeTagDirectory function. Accessible chromatin peaks were called for mapped paired reads using HOMER findpeak function (-minDist 150 –region –fdr 0.05). PCA and sample clustering were performed on accessible peaks using DiffBind. Differentially accessible regions (DAR) in either direction were captured using HOMER getDiffererentialPeaks function (-q 0.05). DAR were annotated using the human GTF annotation file (GRCh38.85) and ChIPSeeker with a promoter definition of -1000 bp and +100 bp around the transcriptional start site (TSS). Peaks overlapping 5’UTRs, promoters, first exons and first introns were pooled for functional enrichment of genes.

### Functional Enrichment

For DEG identified from RNA-Seq, comparative functional enrichment was performed in Metascape (16). For both Methyl-Seq and ATAC-Seq, genes with epigenetic signals overlapping either promoter, 5’ UTR, first exon and intron were enriched using Metascape (Fisher p-value <0.05). Biases in TF regulation were identified using genes within a 10 kb window of promoters using ChEA3 using TopRank predictions. Cis regulation of all regions with epigenetic differences was identified using GREAT using the default model of association (Binomial FDR < 0.05).

### Peak Visualization

To generate pileups, BAM files were converted to bedgraph using bedtools genomecov. Bedgraphs were converted to bigwig files using UCSC genome browser’s bedGraphToBigWig script. Normalized Bigwig files were used for visualization of data on the WashU Epigenome browser.

## Results

### UCB CD4 T Cells From Babies Born to Mothers With Obesity Respond Poorly to Stimulation

**Figure 1:**
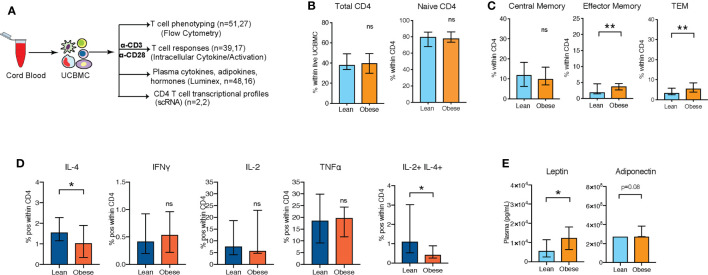
UCB CD4 T cells from babies born to mothers respond poorly to polyclonal T cell activation **(A)** Experimental design: UCBMC from two independent cohorts of babies born to lean mothers and mothers with obesity were phenotyped and stimulated with CD3/CD28 dynabeads. Cytokine responses and activation markers were measured using flow cytometry. Plasma cytokines, chemokines, growth factors, metabolic hormones, and adipokines were measured using luminex. **(B)** Percentage of total CD4+ T cells within UCBMC and naïve cells within CD4+ T cells (n=51, 27) **(C)** Relative abundance of memory subpopulations within CD4+ T cells (n=51, 27). **(D)** Percent IL-4+, IFNγ+, IL-2+, TNFα+ CD4+ T cells, and IL-2+IL-4+ CD4+ Tcells in response to CD3/CD28 stimulation. Error bars show medians with interquartile range (IQR) (n=39,17) **(E)** UCB plasma levels of leptin and adiponectin with maternal obesity (n=48,16). (p-values: * - p < 0.05; ** - p < 0.01).

We recently reported reduced IL-4 responses by UCB CD4+ T cells obtained from babies born to mothers with obesity following overnight stimulation of umbilical cord blood mononuclear cells (UCBMC) with CD3/CD28, while no differences were observed in cord blood CD8+ T cell responses (13). To confirm this phenotype, we repeated the experiment using cord blood samples obtained from two independent cohorts of cord blood samples from babies born to 51 lean (BMI 17-24.9, lean group) and 27 mothers with obesity (BMI 30-50, obese group) (**Figure 1A**). Although, we observed no differences in UCB white blood cell counts, lymphocyte numbers (**Supplementary Figure 1A**) or total CD4+ T cell frequencies within UCBMC (**Supplementary Figure 1A** and **Figure 1B**), pregravid obesity was associated with redistribution of CD4 T cell subsets (**Supplementary Figure 1B** and **Figure 1C**). Specifically, we observed a statistically significant increase in transitional effector memory (TEM) (**Supplementary Figure 1B** and **Figure 1C**) and effector memory CD4 T cells (**Supplementary Figure 1B** and **Figure 1C**) that was accompanied by a slight reduction in central memory (CM) CD4 T cells (**Supplementary Figure 1B** and **Figure 1C**).

Next, we stimulated UCBMC with anti-CD3/CD28 dynabeads and measured intracellular cytokine responses by flow cytometry (**Figure 1A** and **Supplementary Figure 1C**). As previously reported (13), fewer IL-4+ CD4 T cells were observed, while no differences were observed in Th1 responses (**Figure 1D**). Additionally, frequency of polyfunctional T cells expressing both IL-4 and IL-2 was significantly reduced with maternal obesity (**Figure 1D**). We next asked if dampened UCB CD4 T cell responses with maternal obesity could be linked to shifts in inflammatory cytokine milieu and metabolic hormones in cord blood plasma. Luminex analysis of the plasma (**Figure 1A**) revealed a significant elevation in circulating leptin and adiponectin levels (**Figure 1E**) but no differences in insulin or resistin (**Supplementary Figures 1D, E**). Furthermore, we observed no differences in plasma levels of key cytokines, chemokines, or growth factors (**Supplementary Figure 1F**).

### Single Cell RNA Sequencing Reveals Rewiring of UCB CD4+ T Cell Transcriptome With Maternal Obesity

To test if maternal obesity reprograms resting cord blood CD4+ T cells, we performed droplet-based single cell RNA sequencing (scRNA-Seq) of UCBMC from babies born to lean mothers (n=2) and mothers with obesity (n=2). Samples were selected from an independent cohort stratified by maternal BMI. Principal Component Analysis (PCA) and UMAP analysis of all 4 samples revealed all expected major immune cell subsets (T, B, NK, myeloid, and erythroid cells) (**Supplementary Figure 2A**). CD4 T cells were identified based on the expression of CD3D (**Supplementary Figure 2B**) and IL7R (**Supplementary Figure 2C**) but lack of CD8A (**Supplementary Figure 2D**) and constituted the largest cluster (**Supplementary Figure 2A**).

**Figure 2:**
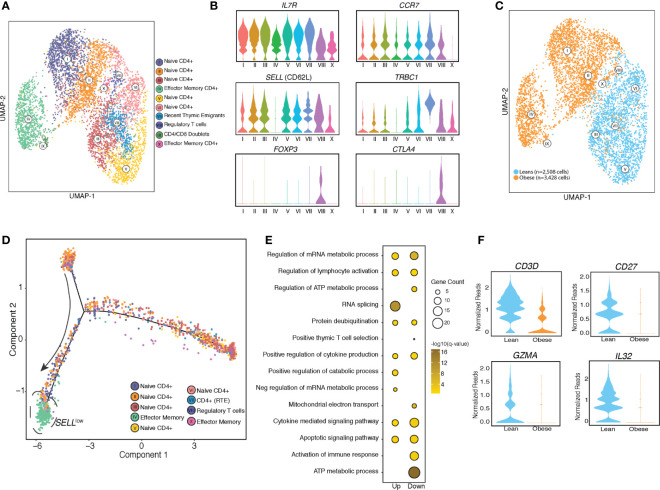
UCB CD4 T cells from babies born to mothers have distinct resting transcriptional profiles. **(A)** UMAP visualization of CD4 T cells clusters from UCBMC single cell profiles of babies born to lean (n=2) and mothers with obesity (n=2) colored by cell state. **(B)** Violin plots of canonical T cell markers associated with naïve T cells, activated T cells, recent thymic emigrants (RTE) and regulatory T cells. **(C)** UMAP visualization of CD4 T cell clusters colored by phenotype – leans colored in blue and obese in orange. **(D)** Trajectory analysis of CD4 T cells, colored by clustered and ordered by pseudotime based on differentially expressed genes identified by monocle. **(E)** Bubble plot representing gene ontology terms enriched from differentially expressed genes (DEG) identified in naïve T cell clusters in obese group relative to the lean group. Size of the bubble represents the number of genes mapping to the term while color indicates level of significance. **(F)** Violin plot showing normalized expression levels of genes downregulated in naïve cord blood CD4+ T cells with maternal obesity.

Re-clustering of concatenated CD4+ T cell clusters led to the identification of 10 clusters (**Figure 2A**). A CD4-CD8 doublet cluster (cluster IX) was removed from all downstream analyses. As expected, most of the UCB CD4+ T cells were naïve cells expressing high levels of *IL7R* and *CCR7* (clusters I-VII) including a small population of recent thymic emigrants (RTE) (cluster VII) expressing high levels of *TRBC1* (**Figure 2B**). Additionally, we identified a small population of regulatory T cells expressing high levels of *FOXP3* (cluster VIII) (**Figure 1D**) and memory T cells expressing low levels of *CCR7* and *SELL* (cluster X) (**Figures 2A, B**). Re-classifying the UMAP based on the group (lean vs. obese) revealed that the naïve T cell compartments from lean and obese groups occupy distinct spaces on the UMAP with clusters I, II, and IV detected in CD4 T cells from the obese group and clusters III, V, VI, and VII detected predominantly in CD4 T cells from the lean groups (**Figure 2C**). Cluster IV which is highly enriched in CD4 T cells from the obese group is composed of memory cells expressing low levels of *SELL* (encodes CD62L) (**Figures 2B, C**). Pseudo temporal analysis and reordering cells on PC1 (**Figure 2D** and **Supplementary Figures 3A, B**) placed cluster IV at the end of the trajectory. Based on down-regulation of *SELL* and relatively low expression of *IL7R* (encoding CD127) relative to other clusters, we conclude that this cluster represents effector memory CD4 T cells.

Differential expression analysis of the naïve T cell clusters revealed that genes downregulated with pregravid obesity enriched to gene ontology (GO) terms associated with immune response, cytokine signaling, and metabolic pathways (**Figure 2E**). Importantly, several genes involved in mounting an effective immune response in lymphocytes (*CD3*, *CD27*, *GZMA*, *IL32*) were downregulated with maternal obesity (**Figure 2F**). On the other hand, genes up-regulated with pregravid obesity enriched to GO terms associated with transcriptional regulation (*JMJD1C*, *CHD1*, *HIF1A*, *TCF25*, and *KLF6*) (**Supplementary Figure 3C**).

### Defects in T Cells Responses in Babies Born to Mothers With Obesity Are Cell Intrinsic

**Figure 3:**
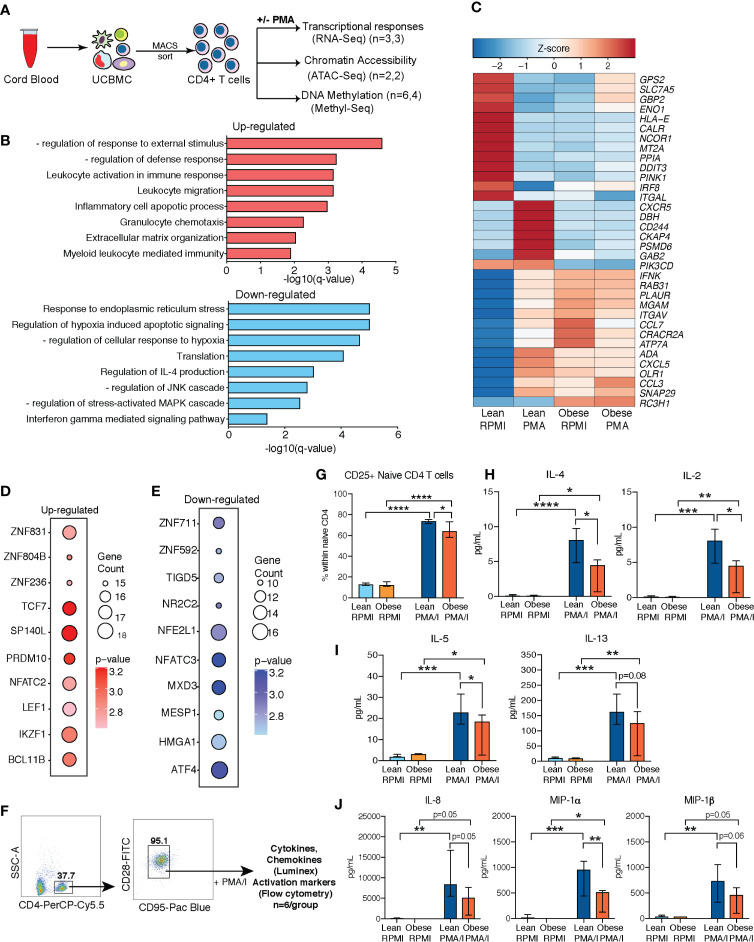
Cell intrinsic defects in cord blood CD4 T cells with maternal obesity. **(A)** Experimental design for testing impact of maternal pregravid obesity on cord blood CD4+ T cells. CD4 T cells were magnetically isolated from UCBMC using MACS and subjected to ATAC-Seq, and MethylSeq (number of samples/group indicated in legend). Purified CD4 T cells were also stimulated with PMA/Ionomycin overnight or left unstimulated. Response was assessed using bulk RNA sequencing. **(B)** Bar graphs of functional enrichment of upregulated (top) and downregulated (bottom) DEG in UCB CD4+ T cells from the lean group following PMA/Ionomycin identified by Metascape. **(C)** Heatmap of representative DEGs detected in UCB CD4 T cells from the lean group that enriched to GO terms “leukocyte activation” and “leukocyte migration”, and “response to ER stress”. **(D, E)** Bubble plots representing TFs regulating **(D)** upregulated (left) and **(E)** downregulated (right) DEG in lean group following PMA stimulation. Size of the bubble represents number of genes and intensity of color represents statistical significance. **(F)** Gating strategy for FACS sorting naïve CD4 T cells from UCBMC. Sorted cells were stimulated with PMA/Ionomycin overnight. Secreted proteins were measured using luminex and T cell activation measured using flow cytometry (n=6/group). **(G)** Relative proportions of naïve CD4 T cells expressing T cell activation marker CD25. **(H–J)** Secreted levels of **(H)** IL-4 and IL-2; **(I)** IL-5 and IL-13), and **(J)** chemokines IL-8, MIP-1α, and MIP-1β following overnight PMA/I stimulation of naïve CD4 T cells from umbilical cord. Error bars represent median with interquartile ranges. (Ordinary one-way ANOVA p-values: * - p < 0.05; ** - p < 0.01; *** - p < 0.001; **** - p < 0.0001).

We next asked if the defect in cytokine responses following T cell activation was intrinsic to cord blood CD4+ T cells or mediated by defective interactions with other UCBMC (**Figure 3A**). To that end, we purified CD4+ cells from a subset of samples using magnetic beads (**Supplementary Figure 4A**) and measured transcriptional responses to activation. RNA-Seq (n=3/group) was performed on cell pellets following overnight culture in the presence or absence of PMA/Ionomycin stimulation (**Figure 3A**). No transcriptional differences were observed between lean and obese group at resting state. In response to PMA stimulation, we observed 461 genes upregulated (log_2_ Fold Change ≥ 1.5 and FDR < 0.05) and 261 genes downregulated (log_2_ Fold Change ≤ -1.5 and FDR < 0.05) in the lean group, but no differentially expressed genes (DEG) were detected in the obese group in response to PMA/ionomycin stimulation. Upregulated DEG detected in lean group enriched to GO terms associated with leukocyte activation and migration (**Figure 3B**), and include genes encoding membrane bound adhesion molecules and receptors (*CXCR5, ITGAV, CD244*) and chemokines (*CCL3, CCL7, CXCL5*) (**Figure 3C**). On the other hand, DEG downregulated with PMA stimulation in the lean group enrich to GO terms associated with regulating immune response (**Figure 3B**) such as “translation”, “response to ER stress” (*CALR*, *DDIT3*, *PINK1, PPIA*), and cytokine signaling pathways (IFNγ and IL-4 signaling) (**Figure 3C**).

Transcription factor (TF) network analysis of DEGs in the lean group shows that up-regulated genes are regulated by TF that mediate effector T cell responses (NFATC2, IKZF1) and T cell differentiation (LEF1, TCF7, BCL11B) (**Figure 3D**) while down-regulated genes are regulated by TF responsive to stress and metabolic changes (ATF4, NFATC3, NFE2L1) (**Figure 3E**).

Next, we asked if increased frequencies of memory T cell subsets with maternal obesity contributed to dampened T cell responses. To address this question, we stimulated sorted naïve CD4 T cells (CD4+CD28+CD95-) with PMA/ionomycin and measured secreted cytokines using Luminex (**Figure 3F** and **Supplementary Table 1**). Surface expression analysis of CD25 and CD69 using flow cytometry revealed significant T cell activation in both groups (**Supplementary Figures 4B, C**); however, CD25 expression was attenuated in the obese group (**Figure 3G**). Furthermore, secreted cytokine measurements revealed dampened levels of both IL-4 and IL-2 (**Figure 3H**) as well as additional Th2 cytokines IL-5 and IL-13 (**Figure 3I**), but not Th1 cytokines (**Supplementary Figure 4D**). Furthermore, no differences were observed in secreted IL-17 (**Supplementary Figure 4D**) or IL-10 (**Supplementary Figure 4D**) levels. Finally, we observed impaired secretion of neonatal CD4 T cell associated chemokines (IL-8, MIP-1α, and MIP-1β) with maternal obesity (**Figure 3J**).

### Cord Blood CD4+ T Cells From Babies Born to Mothers With Obesity Are Epigenetically Poised for Poor Activation

**Figure 4:**
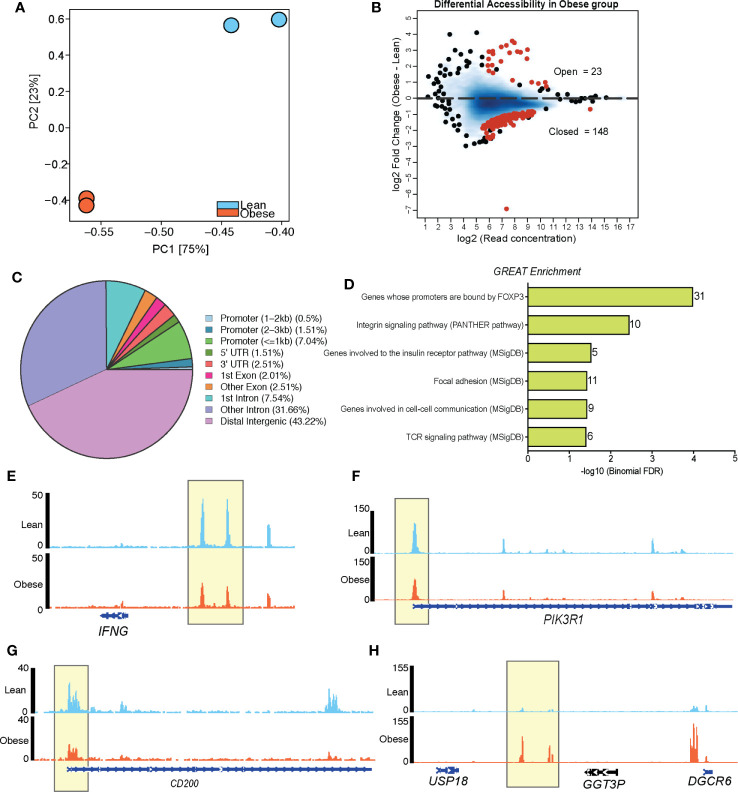
Maternal BMI is associated with distinct chromatin accessibility profiles of loci regulating T cell signaling in cord blood CD4+ cells. **(A)** PCA of chromatin accessibility profiles of resting CD4+ T cells (n=2/group). **(B)** MA plots showing fold changes and coverage of accessible loci. Differentially accessible regions (obese vs leans) are highlighted in red. **(C)** Pie chart showing genomic contexts of DARs in resting cord blood CD4 T cells from obese group relative to lean group. **(D)** Cis-regulatory associations of DARs closed in CD4+ T cells of obese group identified using GREAT. **(E–H)** Pileups of select genes with reduced accessibility **(E–G)** and enhanced accessibility **(H)** in cord blood CD4 T cells from babies born to mothers with obesity.

We next tested if differences in resting epigenome could explain the attenuated UCB CD4 T cell responses to stimulation observed with maternal obesity (**Figure 3A**). We first profiled chromatin states (open vs closed) in purified CD4+ T cells (**Supplementary Figure 4A**) using ATAC-Seq (n=2/group). Bioinformatic analysis revealed significantly different chromatin accessibility profiles between the two groups (**Figure 4A**), with 171 differentially accessible regions (DAR, |log_2_FC| ≥1, FDR <0.05). Most of these DAR (148) were closed in CD4 T cells from the obese group (open in the lean group) (**Figure 4B**) and mapped primarily to distal intergenic regions of the genome (**Figure 4C**). Analysis of the 148 DAR that were closed in UCB CD4 T cells from the obese group using GREAT revealed associations with genes involved in TCR signaling (*IFNG*, *PIK3CB*, *PIK3R1*) (**Figures 4D-F**), cell adhesion (*ITGA2*, *ITGA4*, *ITGA6*, *ITGB1*, *ITGB6*), and cell communication (*NCK2*, *LAMA2*, *INADL*) (**Figure 4D**). Interestingly, a number of the regulatory transcription factor FOXP3 targets (*CD96*, *CD200*, *BTLA*, *ELMO1*, *TLK1*) were closed in UCB CD4 T cells from obese group (**Figures 4D, G**). Finally, loci open in obese group relative to leans (23 regions) mostly mapped to a locus on chromosome 22 involved in DiGeorge syndrome (**Figure 4H**).

### Maternal Obesity Associated Hypomethylation in UCB CD4 T Cells Are Suggestive of Redistribution of Its Subsets

**Figure 5:**
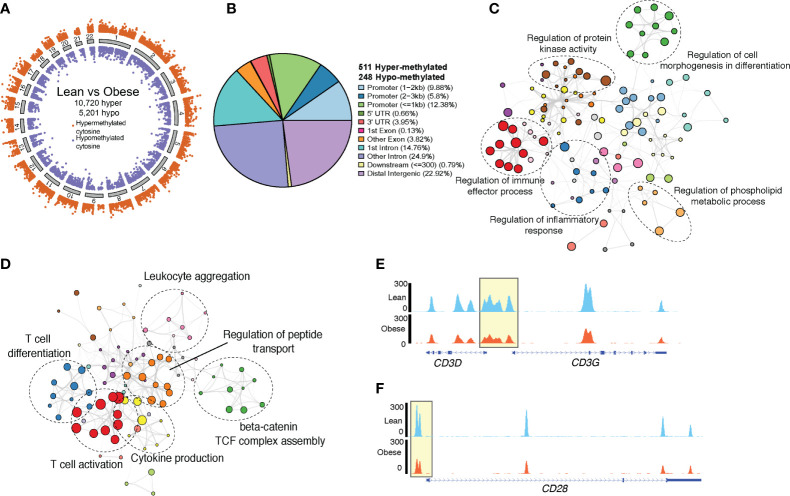
Genome wide methylation changes in UCB CD4+ T cells. **(A)** Ideogram of single base resolution differentially methylated cytosine (DMC) in T cells from obese group relative to lean group (n= 6 lean, 4 obese). Each dot represents a DMC colored by direction of methylation change (orange – hypermethylated in obese vs purple – hypomethylated in obese) and clustered by chromosomes. **(B)** Pie chart showing genomic contexts of differentially methylated loci identified in cord blood CD4 T cells from obese group relative to lean group. **(C)** Network denoting functional enrichment of genes associated with hypermethylated loci in obese group relative to lean group identified by Metascape. **(D)** Network of functional enrichment of genes associated with hypomethylated loci in obese group relative to lean group identified by Metascape. In both networks, dashed ellipse represents a theme of highly related GO terms with individual GO terms as colored bubbles. Size of the bubbles represents the numbers of genes mapped to the term. **(E)** Pileups showing methylation levels of CD3 and **(F)** CD28 loci in lean (top panel, blue) and obese (lower panel, orange) group.

We have previously reported a significant impact of maternal obesity on resting UCB monocyte methylome, which partially explained their poor responses to *ex vivo* LPS stimulation (14). To test if alterations in methylation patterns also contribute to the defect in cord blood CD4 T cell responses, we profiled cytosine methylation at single base resolution using targeted bisulfite sequencing (**Figure 3A**). Differential methylation analysis using MethylKit (1,594,852 bases tested) revealed significant hypermethylation across the genome (10,720 hyper and 5,201 hypomethylated cytosines) in the obese compared to the lean group (**Figure 5A**). We then resolved clusters of differentially methylated cytosine (DMCs) into differentially methylated regions (DMRs) and tested for differences in methylation signals using eDMR. This approach identified 759 differentially methylated regions (DMR) (511 hypermethylated and 248 hypomethylated in the obese relative to the lean group) (**Figure 5B**). Roughly a quarter of these regions overlapped broadly defined 5’ regulatory region and another quarter mapped to distal intergenic regions (**Figure 5B**). Genes regulated by promoters, first exons, and first introns regions that are hypermethylated in the obese group were primarily involved in regulation of inflammatory responses (**Figure 5C**). This list included several myeloid signaling molecules (*CD180*, *FCER1G*, *IFNGR2*, *NOD2*, *NLRP3*) (**Supplementary Figure 5A**), which are traditionally under strong methylation control in T cells as well as chromatin-associated factors (*BRD4*, *CHRAC1*, *CECR2*) (**Supplementary Figure 5A**). Finally, functional enrichment of intergenic loci hypermethylated with maternal pregravid obesity revealed over-representation of bacterial/viral sensing and inflammation pathways (**Supplementary Figure 5B**).

Genes within the vicinity of the 248 hypomethylated loci in cord blood CD4+ T cells from obese group relative to the leans (**Figure 5B**) were involved in T cell activation and differentiation (*CD3G*, *CD28*, *TESPA1*, *SATB1*, *LTB*, *TXK*) and cytokine production (*CD3*, *NFTAC1*, *PRKCZ*, *SYK*, *TNFAIP3*) (**Figure 5D**). Importantly, we report loss of methylation in regulatory loci of both *CD3* and *CD28* with maternal obesity (**Figures 5E, F**). Additionally, hypomethylated DMRs overlapping distal intergenic regions were associated with T cell activation and metabolism (**Supplementary Figure 5C**). Finally, transcription factor analysis of hypomethylated DMRs suggested over-representation of binding sites for TFs associated with memory development (NFATC2, RUNX3, TCF7) (**Supplementary Figure 5D**) and T cell activation (STAT4). Hypermethylated DMRs, however, contained binding sites for several myeloid TFs (CEBPE, CEBPB, NFE2) (**Supplementary Figure 5E**).

## Discussion

It is becoming increasingly evident that *in utero* exposures to maternal factors such as infection, stress, and obesity can alter fetal development, resulting in adverse outcomes that often persist into adulthood (3). Such exposures during critical windows of fetal immune development have been linked to increased incidence of several infectious and chronic diseases such as asthma (17), cardiovascular (18) and metabolic disease (3). Indeed, data from Danish and Northern Finland Birth cohorts suggest that maternal BMI is strongly associated with development of asthma and wheezing in children (19) and adolescents (20). Furthermore, data collected from epidemiological studies and experimental models of HFD induced maternal obesity collectively indicate poor infectious disease outcomes in the offspring (1, 2, 10, 17, 21), suggesting a dysregulated development of the fetal immune system (13). Indeed, our recent studies documented a reduced capacity of cord blood monocytes to produce cytokines and chemokines following LPS stimulation (14). This defect was cell intrinsic and partially mediated by alterations in the DNA methylation landscape (14). In addition to defects in myeloid cells, we also reported reduced frequency of cord blood CD4+ T cells, and reduced IL-4 production in response to ex vivo CD3/CD28 stimulation (13). T cells in human newborns are primarily naïve and mount a Th2 dominant memory response. However, these cells are mature, functional, and poised to sustain effector responses to antigens post birth (22).

In this study, we confirm and extend the phenotype of defective Th2 responses in babies born to lean and obese mothers using two new independent cohorts. We report increased relative frequency of transitional effector memory and effector memory CD4 T cells in cord blood of babies born to mothers with obesity. Their increased numbers in obese group is suggestive of possible antigenic exposure in utero or increased homeostatic proliferation (23). Cord blood T cells have high turnover rates and been shown to proliferate during early fetal life (24). This antigen independent proliferation, however, is regulated by the diversity of memory cell repertoire (25). Whether maternal obesity alters fetal T cell repertoire diversity remains to be tested.

An increase in memory CD4 T cells would be expected to correlate with enhanced *ex vivo* responses in the obese group. Surprisingly, and in agreement with our earlier studies (13), we report significant reduction in production of canonical Th2 (IL-4) cytokines following CD3/CD28 stimulation. RNA-Seq analysis after PMA/ionomycin stimulation suggests a failure to activate transcriptional signatures of immune activation and migration in UCB CD4 T cells from the obese group. This dampened Th2 response was intrinsic to naïve CD4 T cells. Indeed, when stimulated with PMA/ionomycin, naïve CD4 T cells from babies born to obese mothers were less activated and secreted lower levels of IL-2, Th2 cytokines (IL-4, IL-5, and IL-13), and chemokines (MIP-1α, MIP-1β, and IL-8). These findings suggest potential maladaptive T cell polarization and impaired recruitment of innate immune cells following T cell activation.

Poor cytokine and chemokine responses might in turn results in inadequate anti-microbial immunity. Indeed, studies in rodent models of maternal obesity have demonstrated worse outcomes in response to bacterial and viral infections (1, 10). More importantly, clinical studies showed that babies born to mothers with high BMI are at a greater risk of acquiring bacterial infections requiring admission to the neonatal intensive care unit (5, 26). Intriguingly, children born to mothers with obesity are also more prone to developing allergies, wheezing, and asthma later in life (19, 27–29), which are conditions associated with hyper-reactive Th2 responses. Although our data show reduced Th2 responses by UCB CD4 T cells, additional changes could occur during early childhood leading to disrupted T cell polarization later in life. Moreover, tissue resident CD4 T cells could behave differently than circulating T cells. Therefore, future research should investigate the evolution of postnatal memory T cell responses in children born to obese mothers both in circulation and tissue.

Maternal obesity has been previously shown to alter DNA methylation profiles of offspring liver, heart (30), and cord blood (31) cells. We therefore tested epigenetic adaptations of offspring CD4 T cells in response to pregravid obesity using bisulfite sequencing and chromatin accessibility profiling. Specifically, loci associated with T cell activation (*CD3*, *CD28*) were hypomethylated in cord blood CD4 T cells from babies born to mothers with obesity. This observation strongly supported the presence of higher numbers of memory CD4 T cells in the obese group. Indeed, transition of naïve T cells to central memory and effector memory phenotypes has been shown to result in concomitant loss of methylation in genes encoding surface markers and transcription factors associated with memory development (32). First, we observed loss of methylation over several memory T cell associated loci (RUNX3 and NFATC2 associated genes). Second, we observed hypermethylation (intronic and upstream intergenic) of *FOXP1*, a checkpoint regulator that serves as a “naïve-keeping” factor and that is methylated during the transition between naïve and memory phenotype (32). Interestingly, changes in methylation levels in a handful of genes associated with T cell activation (*CD3D*, *CD3G*, *RAC2*, *LTB*, *IL32*) and survival (*SATB1*, *TNFAIP3*) were consistent with their reduced expression with maternal obesity. Taken together, our observations link the methylation changes associated with maternal obesity in cord blood CD4+ T cells with both increased frequency of memory subset and changes in their activation potential.

Several studies have demonstrated the critical role of chromatin remodeling over the course of T cell differentiation and activation (32); therefore, we investigated the impact of pregravid maternal obesity on chromatin accessibility profile in cord blood CD4 T cells that explained poor *ex vivo* responses. Our analysis revealed significant closing of loci associated with TCR signaling (*PDE4B*, *PDE4D*, *PDE7B*) and cell communication (*ITGA2*, *ITGA6*, *ITGB6*, *ITM2B*). Interestingly, *IFNG* promoter, which normally undergoes significant chromatin opening with T cell activation, was significantly closed in the obese group. Finally, a number of closed loci were associated with insulin and PI3 kinase signaling (*IRS1*, *PARD3*, *PDK1*, *PIK3R1*) suggesting potential reprogramming of T cell metabolism. Interestingly, closed DARs also overlapped a number of FOXP3 target genes that are highly expressed in regulatory T cells (*BTLA*, *CD200*, *CD96*, *ITPR2*), which could potentially result in a reduction in Treg frequency or regulatory functions. Although we did not address this question in this study, previous studies in animal models of maternal pregravid obesity have reported a significant reduction in colonic and spleen Tregs in offspring in line with increased risk of autoimmune disease (2). Finally, we observed no chromatin remodeling near IL-4 promoter loci, suggesting additional signaling mechanisms in regulating poor Th2 responses with maternal obesity. While we observed limited overlap between chromatin accessibility and gene expression changes in resting CD4 T cells, genes important for TCR signaling were less accessible, consistent with down-regulation of lymphocyte activation genes in resting UCB CD4 T cells with maternal obesity.

As with any human study, there were some limitations. Despite the large variability in T cell responses and phenotypes, we were able to observe a clear defect in Th2 responses in UCB CD4 T cells. A major limitation of this study was the relatively small sample size for genomic experiments and lack of matched samples across multiple experiments due to the limited volumes of cord blood samples we were able to access. Finally, given their low frequencies in cord blood, we were unable to sort enough memory T cells to perform functional assays. Nevertheless, our analysis indicates that significant changes in epigenome of resting UCB CD4 can be linked to their phenotypic and functional defects with maternal obesity. Taken together with our previous studies in UCB monocytes (14), these results identify a potential explanation for the increased susceptibility to infectious agents in offspring of mothers with obesity.

The precise mechanisms underlying cellular programming events in CD4 T cells remain poorly understood. Recent work in rodent models of HFD-induced obesity links dietary fat and metabolic stress with biased memory CD4 T cell differentiation in adipose and vascular tissue in a PI3K-Akt dependent manner (33). Given these observations, we could speculate similar mechanisms drive changes in offspring CD4 T cells during fetal development. Additionally, changes in T cell development/maturation in the cord blood with maternal obesity could explain the phenotypic and functional defects we describe in this study. Indeed, studies in rodents have demonstrated that maternal high fat diet and obesity compromises fetal hematopoiesis, skewing immune cells towards more mature myeloid and lymphoid cells in early gestational fetal liver (34).

Maternal obesogenic environment has been shown to change the maternal systemic environment which could alter the cord blood plasma cytokine milieu. We and others have shown that obesity during pregnancy presents as a state of chronic low-grade inflammation, with elevated levels of insulin, leptin, adiponectin, CRP, IL-6, and chemokines such as IL-8 (35). However, very little evidence supports the transfer of cytokines and chemokines across the placental barrier (36). Indeed, our analysis of cord blood plasma cytokines and chemokines revealed no differences with maternal obesity. Interestingly, both leptin and adiponectin levels were elevated in cord blood of babies born to obese mothers. While leptin promotes T cell activation and cytokine production (37), adiponectin has been shown to regulate Th1 and Th17 responses in naïve T cells (38). Therefore, future studies will need to focus on the potential mechanisms by which these metabolic hormones could impact CD4 T cells maturation and function. Moreover, extrinsic factors such as suppressive myeloid cells could contribute to the re-wiring of fetal/neonatal CD4 T cells resulting in altered responses following antigen encounter. However, the exact contributions of this putative mechanisms to the dampened Th2 responses reported here remain to be defined.

## Data Availability Statement

The datasets supporting the conclusions of this article are available on NCBI’s Sequence Read Archive (BioProject IDs: PRJNA690128 and PRJNA690532).

## Ethics Statement

This research project was approved by the Institutional Ethics Review Boards (IRB) of Oregon Health and Science University, University of California Riverside, and University of California Irvine. The patients/participants provided their written informed consent to participate in this study.

## Author Contributions

SS, NM, and IM conceived and designed the experiments. SS, RW, NM, and AJ performed the experiments. SS and NM analyzed the data. SS and IM wrote the paper with input from NM. All authors contributed to the article and approved the submitted version.

## Funding

This work was supported by grants from the National Institutes of Health 1K23HD06952 (NM), R03AI112808 (IM), 1R01AI142841 (IM), and 1R01AI145910 (IM).

## Conflict of Interest

The authors declare that the research was conducted in the absence of any commercial or financial relationships that could be construed as a potential conflict of interest.

## Supplementary Material

Supplementary Figure 1 Phenotypic changes in UCB CD4 T cells with maternal obesity. **(A)** Numbers of white blood cells (WBC) and lymphocytes measured in whole blood (n= 51 lean, 27 obese). **(B)** Gating strategy for characterization of total CD4+ T cells and their subsets. **(C)** Gating strategy for recording intracellular readouts of T cell cytokines in CD4 T cells in UCBMC following overnight CD3/CD28 stimulation. (D-E) Plasma levels of metabolic hormones **(D)** insulin and PYY; and **(E)** adipokines adipsin, resistin, and lipocalin. **(F)** Bubble plot representing changes in immune mediator levels in plasma with gestation. Size of the bubble represents median values of analytes in pg/mL (log10 transformed). The colors are scaled based on p-values from unpaired t-test (-log10).

Supplementary Figure 2 Single cell profile of immune cells in cord blood mononuclear cells from term deliveries. **(A)** UMAP visualization of UCBMC from babies born to lean mothers (n=2) and mothers with obesity (n=2). Clusters were identified based on canonical transcript markers from previous studies. **(B)** Feature plots of T cell markers *CD3D* **(C)** *IL7R* (CD127 gene) and **(D)** *CD8A*. These markers were used to identify CD4 T cell clusters.

Supplementary Figure 3 Differential profiles of UCB CD4 T cells with maternal obesity. **(A)** Trajectory analysis of CD4 T cells, colored and ordered by pseudotime based on differentially expressed genes identified by monocle. **(B)** Individual clusters ordered by Principal Component 1. **(C)** Violin plot showing normalized expression levels of epigenetic regulators and transcription factors up-regulated in naïve cord blood CD4+ T cells with maternal obesity.

Supplementary Figure 4 Cell intrinsic defect in T cell responses. **(A)** Representative histogram illustrating purity of cord blood CD4+ T cells following magnetic bead separation. **(B)** Representative gating strategy for measuring activation of naïve UCB CD4 T cells using surface markers. **(C)** Relative frequencies of CD69 expressing naïve CD4+ T cells. **(D)** Bar graphs comparing secreted Th1 cytokines TNFα and IFNγ; IL-17, and IL-10 following overnight stimulation of sorted naïve CD4 T cells with PMA/ionomycin. Error bars represent median values (pg/mL) and interquartile ranges. (Ordinary one-way ANOVA p-values: * - p<0.05; ** - p<0.01; **** - p<0.0001).

Supplementary Figure 5 Epigenetic changes in cord blood CD4 with maternal obesity. **(A)** Volcano plot of DMRs overlapping vicinity of promoters. Genes associated with the loci are annotated with colors depending on the genomic context. **(B)** Cis-regulatory associations of DMRs hypermethylated and **(C)** hypomethylated in CD4+ T cells of obese group identified using GREAT. **(D)** Bubble plots representing number of hypomethylated and **(E)** hypermethylated DMRs overlapping genes regulated by transcription factors predicted by ChEA3. Size of the bubble represents the numbers of genes regulated by each transcription factor.
