# Supplementary figures and images for: Phenotypic and Epigenetic Adaptations of Cord Blood CD4+ T Cells to Maternal Obesity
Source: Front Immunol. 2021 Apr 12;12:617592. doi: 10.3389/fimmu.2021.617592 (PMC8071865; doi:10.3389/fimmu.2021.617592)

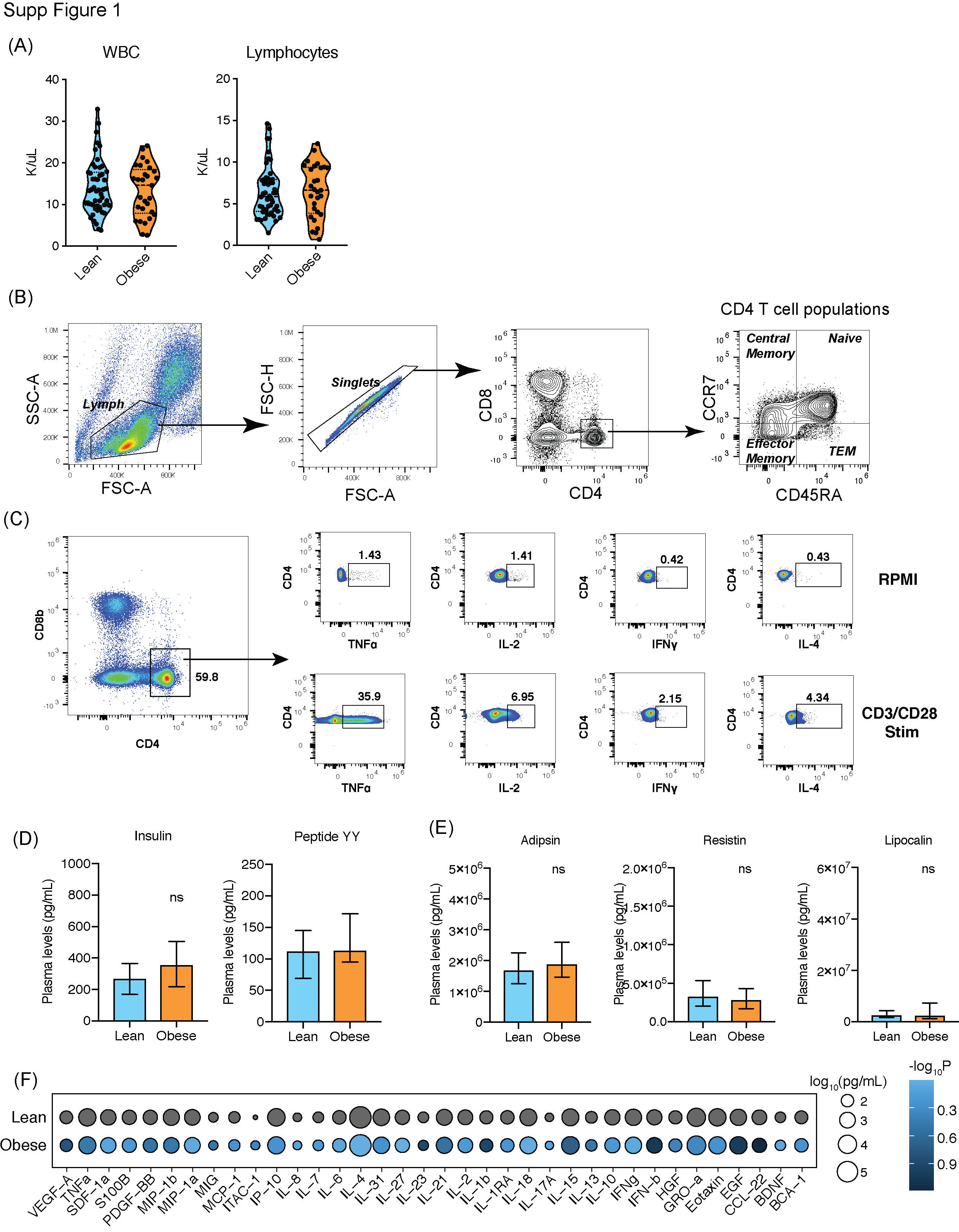

Supplement: Supplementary Figure 1 — Phenotypic changes in UCB CD4 T cells with maternal obesity. (A) Numbers of white blood cells (WBC) and lymphocytes measured in whole blood (n= 51 lean, 27 obese). (B) Gating strategy for characterization of total CD4+ T cells and their subsets. (C) Gating strategy for recording intracellular readouts of T cell cytokines in CD4 T cells in UCBMC following overnight CD3/CD28 stimulation. (D-E) Plasma levels of metabolic hormones (D) insulin and PYY; and (E) adipokines adipsin, resistin, and lipocalin. (F) Bubble plot representing changes in immune mediator levels in plasma with gestation. Size of the bubble represents median values of analytes in pg/mL (log10 transformed). The colors are scaled based on p-values from unpaired t-test (-log10). [file Image_1.tif]

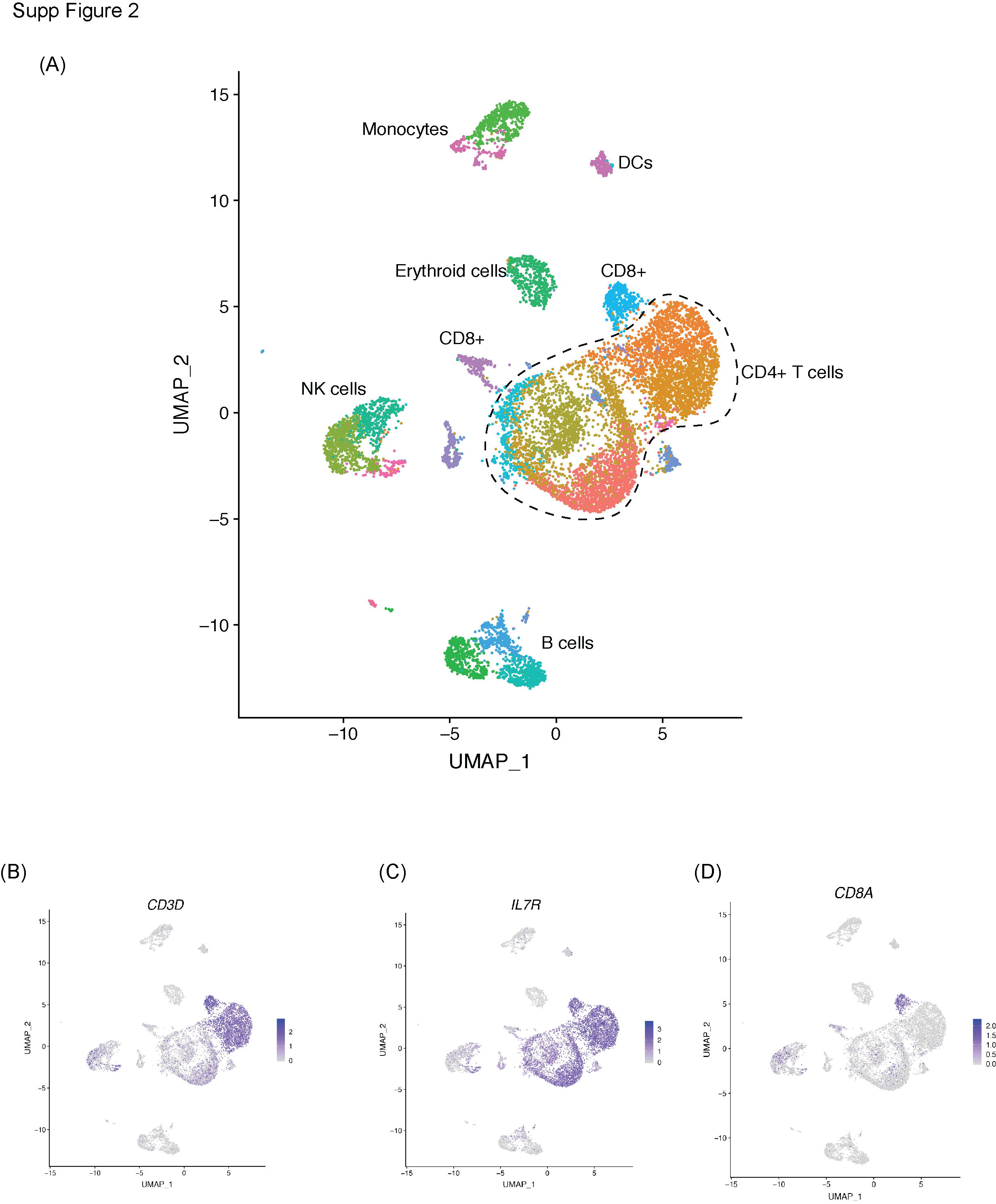

Supplement: Supplementary Figure 2 — Single cell profile of immune cells in cord blood mononuclear cells from term deliveries. (A) UMAP visualization of UCBMC from babies born to lean mothers (n=2) and mothers with obesity (n=2). Clusters were identified based on canonical transcript markers from previous studies. (B) Feature plots of T cell markers CD3D (C) IL7R (CD127 gene) and (D) CD8A. These markers were used to identify CD4 T cell clusters. [file Image_2.tif]

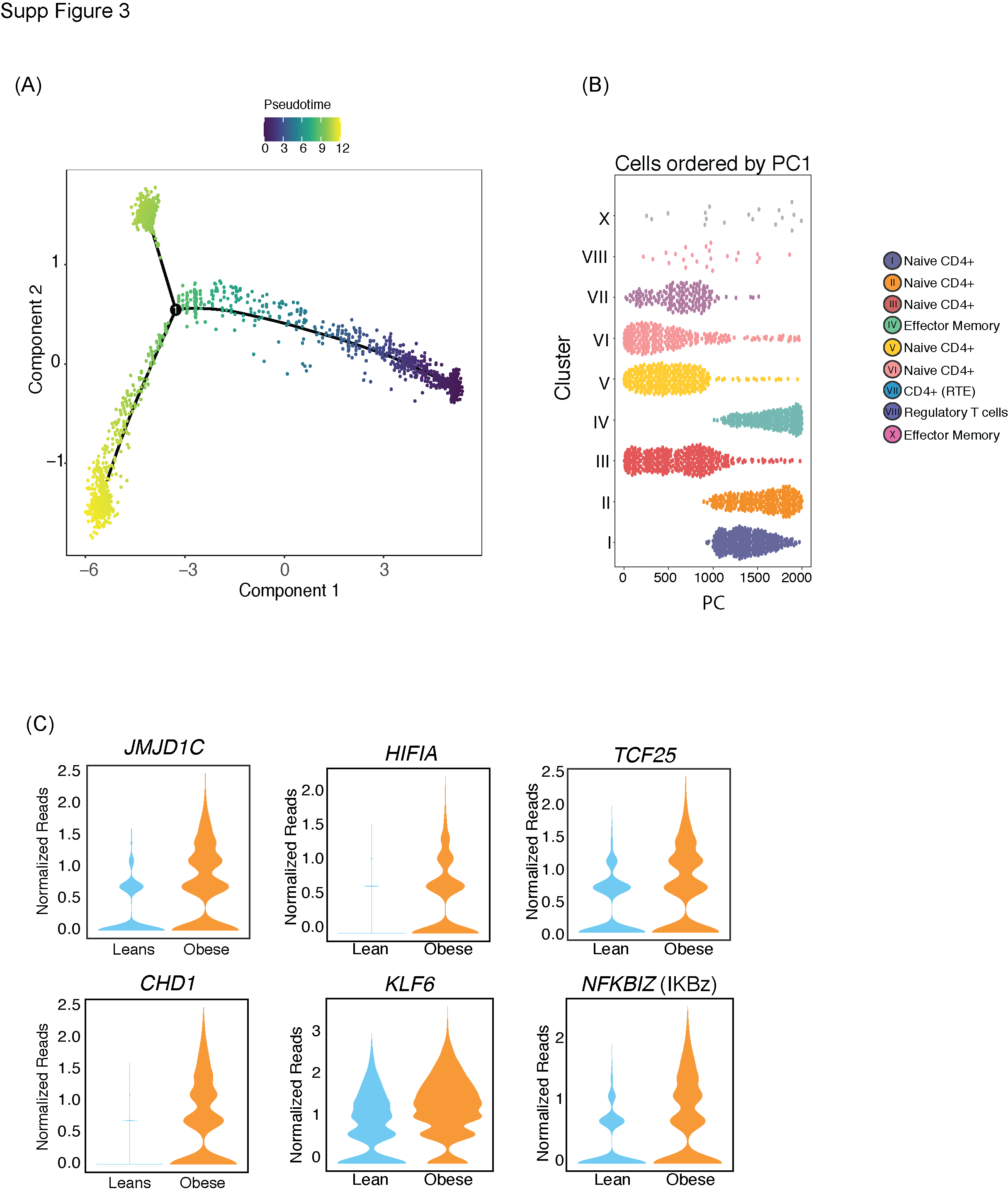

Supplement: Supplementary Figure 3 — Differential profiles of UCB CD4 T cells with maternal obesity. (A) Trajectory analysis of CD4 T cells, colored and ordered by pseudotime based on differentially expressed genes identified by monocle. (B) Individual clusters ordered by Principal Component 1. (C) Violin plot showing normalized expression levels of epigenetic regulators and transcription factors up-regulated in naïve cord blood CD4+ T cells with maternal obesity. [file Image_3.tif]

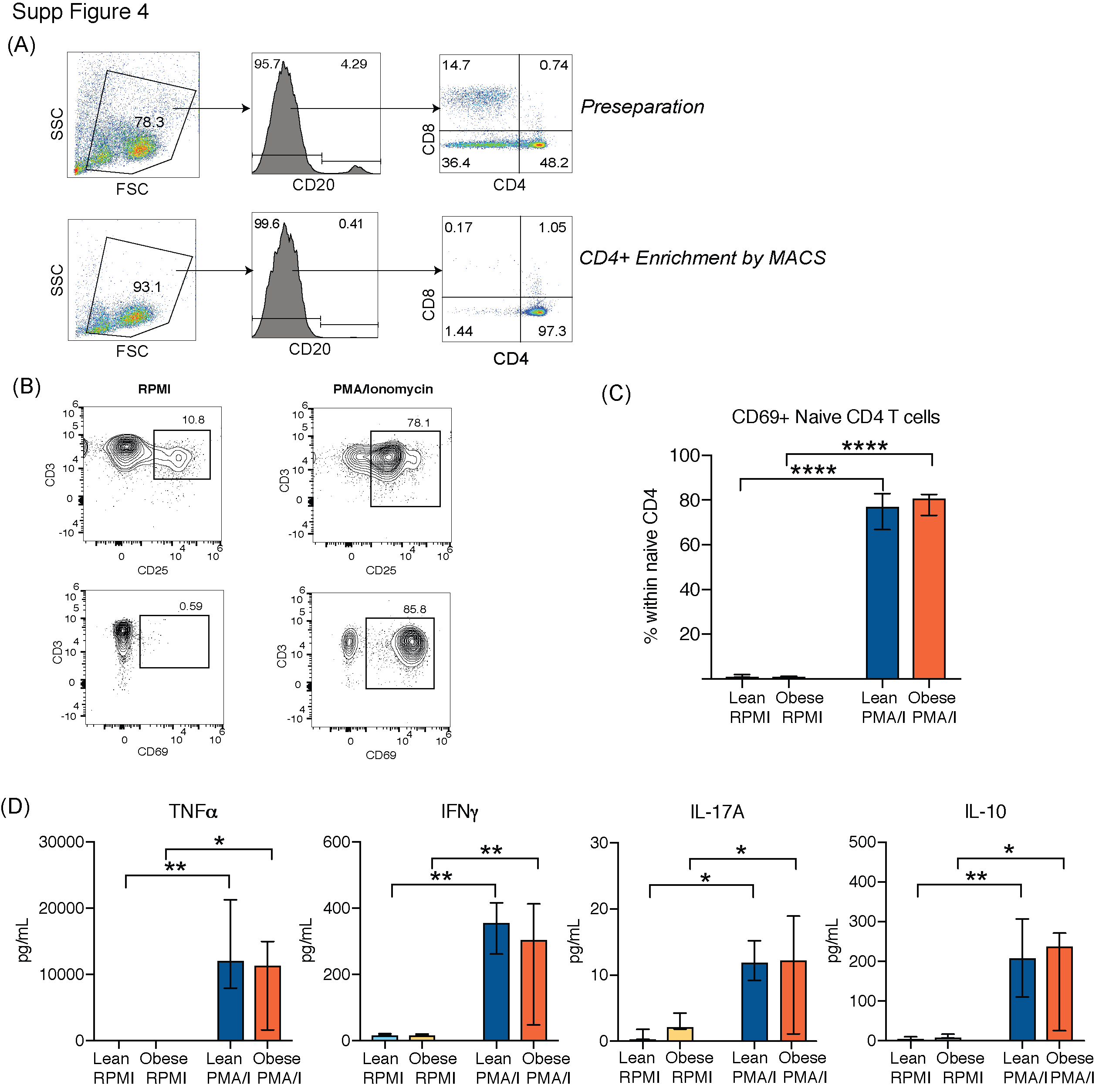

Supplement: Supplementary Figure 4 — Cell intrinsic defect in T cell responses. (A) Representative histogram illustrating purity of cord blood CD4+ T cells following magnetic bead separation. (B) Representative gating strategy for measuring activation of naïve UCB CD4 T cells using surface markers. (C) Relative frequencies of CD69 expressing naïve CD4+ T cells. (D) Bar graphs comparing secreted Th1 cytokines TNFα and IFNγ; IL-17, and IL-10 following overnight stimulation of sorted naïve CD4 T cells with PMA/ionomycin. Error bars represent median values (pg/mL) and interquartile ranges. (Ordinary one-way ANOVA p-values: * - p<0.05; ** - p<0.01; **** - p<0.0001). [file Image_4.tif]

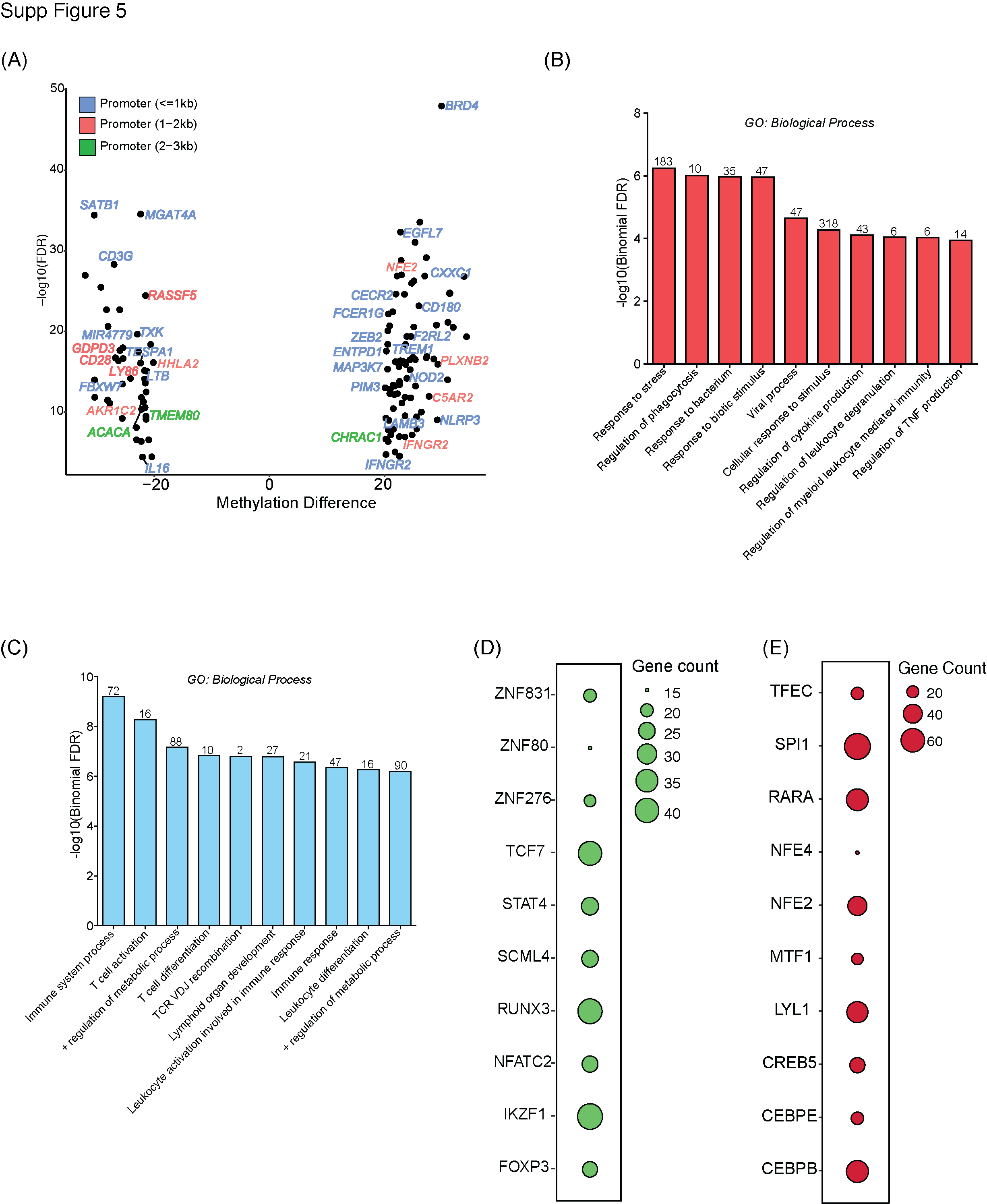

Supplement: Supplementary Figure 5 — Epigenetic changes in cord blood CD4 with maternal obesity. (A) Volcano plot of DMRs overlapping vicinity of promoters. Genes associated with the loci are annotated with colors depending on the genomic context. (B) Cis-regulatory associations of DMRs hypermethylated and (C) hypomethylated in CD4+ T cells of obese group identified using GREAT. (D) Bubble plots representing number of hypomethylated and (E) hypermethylated DMRs overlapping genes regulated by transcription factors predicted by ChEA3. Size of the bubble represents the numbers of genes regulated by each transcription factor. [file Image_5.tif]
